# Developing a Core Outcome Set for Clinical Trials of Chinese Medicine for Hyperlipidemia

**DOI:** 10.3389/fphar.2022.847101

**Published:** 2022-05-02

**Authors:** Geng Li, Ruxue Han, Mingjun Lin, Zehuai Wen, Xiankun Chen

**Affiliations:** ^1^ The Second Affiliated Hospital of Guangzhou University of Chinese Medicine, Guangzhou, China; ^2^ Key Unit of Methodology in Clinical Research, Guangdong Provincial Hospital of Chinese Medicine, Guangzhou, China; ^3^ Foshan Hospital of Traditional Chinese Medicine, Foshan, China; ^4^ Guangzhou Nansha Hospital of TCM, Guangzhou, China; ^5^ State Key Laboratory of Dampness Syndrome of Chinese Medicine, The Second Affiliated Hospital of Guangzhou University of Chinese Medicine, Guangzhou, China; ^6^ Guangdong Provincial Key Laboratory of Clinical Research on Traditional Chinese Medicine Syndrome, Guangzhou, China; ^7^ Department of Global Public Health, Health Systems and Policy, Karolinska Institute, Stockholm, Sweden

**Keywords:** hyperlipidemia, core outcome set, Chinese medicine, systematic review, delphi survey

## Abstract

**Background:** Chinese medicine (CM) is widely used for treating hyperlipidemias, especially in China. However, the heterogeneity of outcomes measured and reported across trials exacerbates the obstacles of evidence synthesis and effectiveness comparison. In this study, we develop a core outcome set (COS) for CM clinical trials for hyperlipidemia (COS-CM-Hyperlipidemia) to tackle the outcome issues.

**Methods:** We generated candidate outcomes through a systematic review of interventional and observational studies of Chinese medicine for hyperlipidemias. The comprehensive search strategy was employed. Study selection and data collection were independently done by two researchers. We searched clinical trial registry platform to supplement the outcomes list extracted by systematic review. Then, we conducted a three-round Delphi survey. The stakeholders were hyperlipidemia patients, clinicians or researchers, in either CM/integrated Chinese or Western medicine, clinical pharmacy, clinical epidemiology or statisticians, or editors of important relevant journals and an ethicist. They used a 9-point Likert scale to determine how important they felt each outcome was in determining treatment success. A consensus meeting was held to confirm the final COS, based on the Delphi survey results.

**Results:** We identified a total of 433 outcomes from 3,547 articles, and 28 outcomes from 367 registered trials. After standardization, we selected 71 outcomes to develop a preliminary outcome list for further consensus. After three Delphi survey rounds and one consensus meeting, the most important outcomes were determined for COS-CM-Hyperlipidemia. It included cardiovascular events, low-density lipoprotein cholesterol, risk of cardiovascular disease, total cholesterol, carotid intima-media thickness, high-density lipoprotein cholesterol, triglycerides, cerebrovascular events, adverse drug reactions and patient-reported symptoms.

**Conclusion:** COS-CM-Hyperlipidemia may improve outcome reporting consistency in clinical trials. Further work is needed to explore the optimal methods for measuring these outcomes.

**Registration:** The Core Outcome Measures in Effectiveness Trials Initiative (COMET): http://www.cometinitiative.org/studies/details/983. Registered on 25 April 2017.

## 1 Introduction

Cardiovascular diseases (CVDs) are the leading cause of death globally, taking an estimated 17.9 million lives each year.^1^ People with hyperlipidemia are at roughly twice the risk of developing CVDs as those without (Karr, 2017). High cholesterol (one type of hyperlipidemia) is one of the primary causal risk factors for CVDs and is 1 of 7 critical metrics the American Heart Association has used to define cardiovascular health in adults and children (Virani et al., 2020). According to the 2017 Global Burden of Disease Study (GBD 2017 Risk Factor Collaborators, 2018), of the leading risk factors for global mortality, high low-density lipoprotein cholesterol (LDL-C) remained the fifth-leading risk factor for mortality in both 1990 and 2017, accounting for 4.3 million deaths in 2017 (Virani et al., 2020).

Statins are the cornerstone of hyperlipidemia therapy, in addition to healthy lifestyle interventions (Grundy et al., 2019). Chinese medicine (CM) is widely used for treating hyperlipidemias, especially in China (Liu et al., 2011; Liao et al., 2014; Yao et al., 2016; Wang and Qiu, 2019). Thus, there has been an increasing focus on trials of CM on hyperlipidemias, yet there remains a lack of studies with high methodological quality. Researchers have suggested that this may be attributable to heterogeneity in the outcomes measured and reported across trials (Zhang et al., 2013; Xing et al., 2014). The lack of standardization of hyperlipidemia outcomes exacerbates the obstacles to evidence synthesis and CM effectiveness comparison (Williamson et al., 2017). Developing a core outcome set (COS) is one approach to addressing this lack of quality and standardization.

A COS represents the minimum outcomes that should be measured and reported in all clinical trials for a specific condition to facilitate the comparison and combination of trials while researchers continue to explore other outcomes (Williamson et al., 2017).^2^ CM hyperlipidemia treatment involves CM patterns (syndromes, or zheng in Chinese) which need to be considered by syndrome differentiation according to a patient’s clinical manifestations, including their pulse and tongue. Thus, outcome assessment may deviate from that used in Western medicine (Zhang et al., 2013; Xing et al., 2014; Qiu et al., 2021). Therefore, the aim of this study was to develop a COS for clinical trials of Chinese Medicine hyperlipidemia treatments (COS-CM-Hyperlipidemia).

## 2 Methods

This study has been registered on the Core Outcome Measures in Effectiveness Trials (COMET) website (No. 983)^3^ and the protocol has been published (Li et al., 2019). The conduct of this COS development adhered to the COMET handbook as much as possible (Williamson et al., 2017), and its results have been reported following the Core Outcome Set–STAndards for Reporting (the COS-STAR Statement) (Kirkham et al., 2016).

We established a work group and a study advisor group (SAG), and used group discussion, a Delphi survey and consensus meeting methods sequentially to develop COS-CM-Hyperlipidemia.

Ethical approval was obtained from the ethics committee of Guangdong Provincial Hospital of Chinese Medicine (GPHCM). Informed consent was obtained from all participants. All personal information about potential and enrolled participants will remain confidential.

### 2.1 Participants

#### 2.1.1 Study Advisory Group

We established a SAG with participants from various stakeholder groups to guarantee quality and efficiency in COS-CM-Hyperlipidemia development. The SAG was composed of nine members—two endocrinologists and one cardiovascular expert from GPHCM, two GPHCM outpatients, two methodologists from GPHCM, one cardiovascular clinical trial researcher from GPHCM, one ethicist from the GPHCM ethics committee, and one statistician from GPHCM. The SAG was responsible for confirming the candidate outcome set for the Delphi survey, participating in the consensus meeting, process coordination, as well as data analysis and interpretation.

#### 2.1.2 Delphi Survey Panel Assembly

According to the principle of representativeness and authority, we recruited experts in CM/integrated Chinese and Western medicine, clinical pharmacology, clinical epidemiology and statistics, medical journal editors and patients to participate in the Delphi survey. We expected to select 50 participants using a snowball sampling method. We identified a preliminary list of experts by reviewing the authors of high-impact papers and selected preliminary patients from a pool of GPHCM outpatients. Then, the preliminary stakeholders recommended whomever else they thought should be included as relevant stakeholders.

#### 2.1.3 Consensus Meeting Participants

We adopted the purposeful sampling method to select participants who had completed all three rounds of the Delphi survey and invited at least two representatives from patients, endocrinologists, cardiologists, hyperlipidemia specialist nurses, and hyperlipidemia researchers to the consensus meeting. Additionally, all members of the SAG took part in the consensus meeting.

### 2.2 Information Sources

We conducted a systematic review of literature on studies of CM for hyperlipidemia (Li et al., 2021). Then, we searched three English databases (PubMed, Cochrane Central Register of Controlled Trials (CENTRAL), and Embase) and three Chinese databases (China National Knowledge Infrastructure (CNKI), Chinese BioMedical Database (CBM), and Wanfang Database) in October 2017, with no time restriction. In order to collect comprehensive outcomes, we also searched two clinical trial registries (http://www.chictr.org.cn/index.aspx, https://clinicaltrials.gov/) to retrieve any outcomes used in clinical trials between 1 January 2016, and 1 January 2019.

We included randomized controlled trials, non-randomized controlled trials, case series, case-control, cohort studies, and systematic reviews evaluating CM for hyperlipidemia. Studies were excluded if 1) they lacked either clear diagnosis and effectiveness assessment standards or hyperlipidemia outcome reporting; 2) patients had only been treated by Western medicine.

Then, two reviewers (GL and RH) independently extracted the data, and entered it into a database using EpiData 3.1 (EpiData Association, Denmark). Information included the characteristics of each study (e.g., title, publishing journal, author(s), year of publication, country, authors’ affiliation(s), funding, diagnosis criteria, patient source, type of hyperlipidemia complications, follow-up duration, number of patients who withdrew, intervention details and CM syndrome pattern), study design, treatment, blood lipids, names of outcomes and whether they had been specified as primary or secondary outcomes, definitions of outcomes, time-point and method of outcome measurement and adverse events. Disagreements were resolved by discussion or consulting a third researcher (ZW).

After data extraction, we assigned the outcomes to one of eight domains: 1) mortality-related outcomes, 2) pathological or pathophysiological outcomes, 3) response rate-related outcomes, 4) cardiovascular events, 5) symptoms or function-related outcomes, 6) adverse events or safety-related outcomes, 7) patient-reported outcomes, and 8) resource utilization-related outcomes. In order to generate a candidate outcome set for consensus, the SAG members scored all of the outcomes in each domain to determine whether or not they would be included.

### 2.3 Consensus Process

We conducted a three-round Delphi survey to assess experts’ opinions on the importance of the candidate outcomes. Then we held a consensus meeting, attended by key stakeholders, to finalize COS-CM-Hyperlipidemia.

#### 2.3.1 Delphi Survey

For each topic domain, we sorted candidate items alphabetically. For patient panelists, we presented lay equivalents of each outcome instead of scientific terms (Allin et al., 2016). For some complicated or difficult to comprehend outcomes, we enclosed a definition and explanation in the Delphi survey. In Round 1, participants could suggest outcomes not included in the questionnaire which they felt were important. In Delphi Rounds 2 and 3, outcome scores from the questionnaire assigned in the previous round were presented, and the distribution of each outcome from each stakeholder group was attached.

#### 2.3.2 Scoring Importance of Outcome

To evaluate the outcomes’ importance, we used a 9-point Likert scale, where 1, 2 and 3 meant “unimportant” 4, 5 and 6 meant “important, but not essential”; and 7, 8 and 9 meant “essential” (Guyatt et al., 2011). At the end of each round, we performed data analysis on individual stakeholder groups, and as a whole. After Delphi Round 1, some of the outcomes recommended by the experts would enter the second round after discussion by the SAG. All scored outcomes were included in Delphi Round 2. Outcomes that were scored 7–9 by a majority of the panelists in Round two were retained for Delphi Round 3. After conducting the Delphi Round three analysis, we assigned the outcomes to one of three categories: ‘consensus out’ (≥70% scoring 1 to 3 AND <15% scoring 7 to 9 in each stakeholder group), ‘consensus in’ (<15% scoring 1 to 3 AND ≥ 70% scoring 7 to 9 in each stakeholder group) or ‘without consensus’ (other conditions) (Harman et al., 2013; Hirsch et al., 2016; Williamson et al., 2017).

#### 2.3.3 Consensus Meeting

We reported the results of each Delphi round and the classification of candidate outcomes from the third Delphi round. Outcomes of “consensus in” were considered either “yes” for selected or “no” for not selected. Those voted for by at least 70% of the participants were included in the final COS-CM-Hyperlipidemia. Outcomes scored “consensus out” were excluded, and outcomes of “without consensus” were discussed and re-scored using the same 9-point Likert scale until a final consensus was reached (Allin et al., 2016; Iyengar et al., 2016). The definitions of outcomes were also provided to the expert panel. The same consensus criteria in round three were used at the meeting.

### 2.4 Data Management and Quality Control

This study process was implemented in accordance with the pre-developed protocol following the COMET handbook. We double-checked the data collected during three rounds of the survey process and confirmed the accurate entry and summary. All data from the consensus meeting have been checked through the meeting minutes.

## 3 Results

This study was completed according to the study protocol (Li et al., 2019). One deviation occurred in the data analysis of Delphi Round 2. Since the protocol defined criteria as ‘any outcomes whose median is greater than or equal to 4 (by any stakeholder group) will continue to Delphi Round 3’ being unable to exclude any outcomes, we changed the criteria to ‘outcomes that were scored 7–9 by more than 50% panelists in Round two were retained for Delphi round 3’. The other deviation was that: Due to the COVID-19 outbreak, the face-to-face consensus meeting had to be held as a combination web/in-person meeting.

### 3.1 Outcomes Extracted in the Systematic Review

**FIGURE 1:**
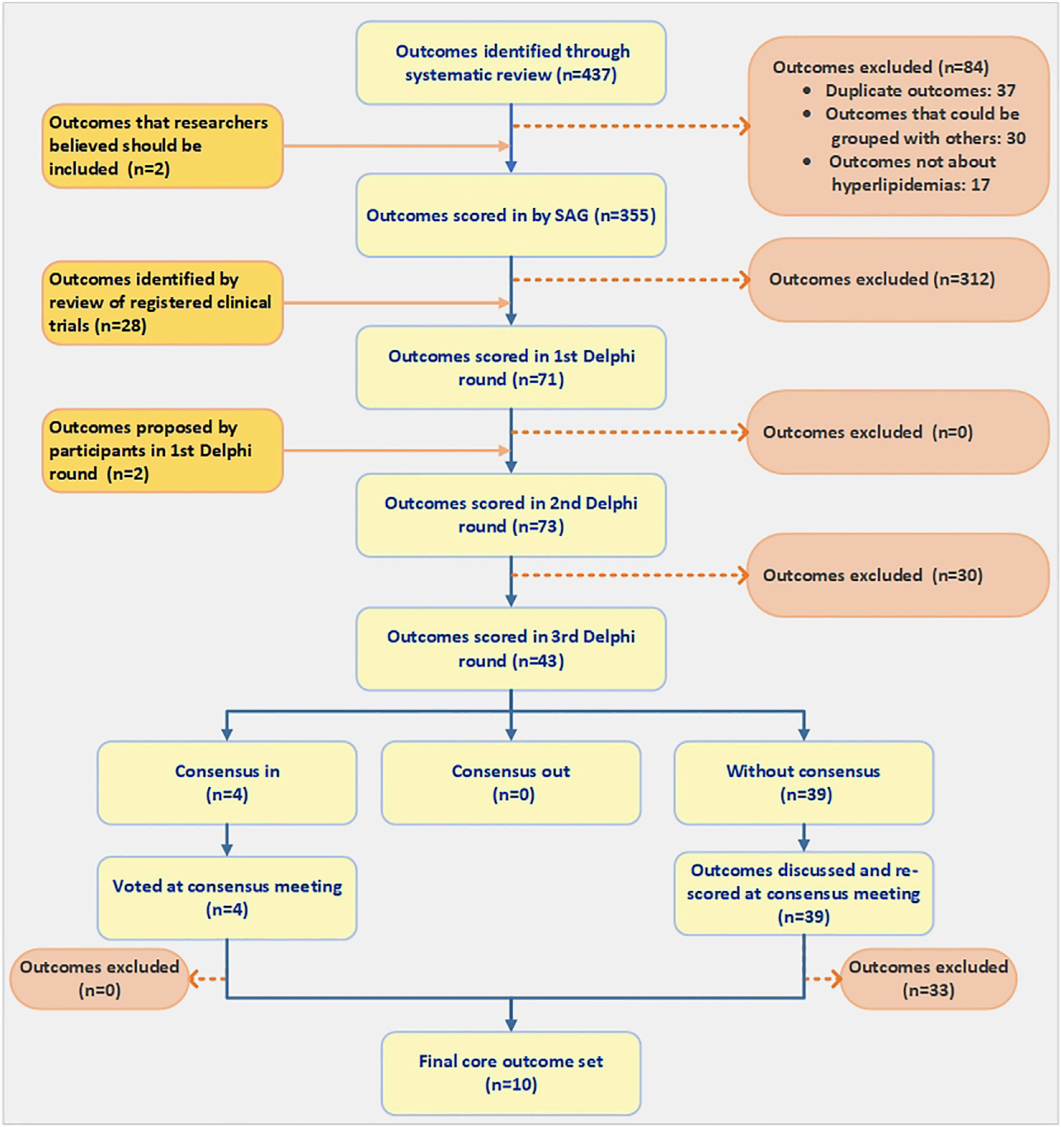
Flow chart of outcome identification and selection.

The systematic review identified 51,905 articles; 3,547 (3,461 in Chinese and 86 in English) were eligible for inclusion following abstract, title, and full-text screening. Supplementary File 1 is the PRISMA flowchart. A total of 433 unique outcomes were identified and reported, which were grouped into eight outcome domains. Researchers reviewed these 433 outcomes, identified 37 duplicates, grouped 25 closely related outcomes, and deleted 18 outcomes that were definitively not about hyperlipidemia. Because this list of outcomes was too long to be used for a Delphi survey, and in order to optimize the Delphi survey, SAG members evaluated the remaining 353 outcomes and two other outcomes that researchers believed should be considered and excluded 312 of them from the candidate outcome set. Additionally, we obtained 367 registered trials on Chinese medicine for hyperlipidemias, and from them, we derived 28 new outcomes. Therefore, a final inventory of 71 outcomes was entered into the first Delphi round (Figure 1) (Supplementary File 2). We also conducted an updated search from November 2017 to November 2020 to identify more new outcomes. Ultimately, we found 19 new outcomes, of which 1 outcome could be grouped with other outcomes, and we deleted the other 18 outcomes after discussion by SAG members (Li et al., 2021). Supplementary File 3 is a list of all 452 original outcomes.

### 3.2 Delphi Survey

Participants’ characteristics for each round are presented in Table 1. A total of ninety experts and patients were invited to participate in the Delphi Round one survey. 70 (75.27%) of them completed the survey and six additional outcomes were suggested by respondents (see Supplementary File 2). All outcomes moved on to the next round. After being assessed by SAG members, two of the six additional outcomes, fatty liver and cerebrovascular events, were included in the second round (*n* = 73) (see Figure 1 and Supplementary File 2).

**TABLE 1:** Participant characteristics across Delphi rounds and consensus meetings.

Stakeholder Group	Round 1 Response Rate (*n* = 70)	Round 2 Response Rate (*n* = 61)	Round 3 Response Rate (*n* = 59)	Consensus meeting 1 (*n* = 19)	Consensus meeting 2 (*n* = 12)
Stakeholder group					
Patients	29	20	18	3	2
Clinicians or researchers in CM/integrated Chinese and Western medicine	27	27	27	9	4
Clinical pharmacy	4	4	4	0	0
Clinical epidemiology	4	4	4	2	4
Statistics	4	4	4	3	1
Editors of important relevant Journals	2	2	2	1	0
Ethicist	0	0	0	1	1
**Sex**					
Male	31	29	27	9	6
Female	39	32	32	10	6
**Academic title**					
Senior	24	24	22	12	8
Intermediate	24	23	23	5	3
Junior	4	4	4	0	1
Prefer not to say	18	10	10	2	0
**Educational background**					
PhD	17	17	17	9	6
Master	19	19	19	7	4
Bachelor	12	11	9	2	1
High school or below	19	12	12	1	1
Prefer not to say	3	2	2	0	0

We invited seventy participants who had completed the Round one survey to re-rate the second round of the Delphi survey, and received 61 (87.14%) responses. The questionnaire listed the outcome scores voted for in the first round, as well as the distribution of each outcome from each stakeholder group. The 43 outcomes which were scored 7–9 by a majority of the panelists moved on to Round 3, while the others were excluded.

We invited the sixty-one participants who had completed Round two to re-rate the third round of the Delphi survey, and received 59 (96.72%) responses. Also, the questionnaire at this stage presented the voting results of the second round, and the distribution of each outcome from each stakeholder group was attached. After analysis, we categorized four outcomes (cardiovascular events, LDL-C, risk of cardiovascular disease and total cholesterol (TC)) as ‘consensus in’, while others were ‘without consensus’ (see Table 2). All outcomes were then discussed at the consensus meeting.

**TABLE 2:** Results of the 3rd round of the Delphi survey.

Consensus Classification	Definition	Outcomes
*Consensus out*	≥70% scoring 1 to 3 AND <15% scoring 7 to 9 in each stakeholder group	
*Without consensus*	Any other conditions	Plaque size, plaque area, number of plaques, atheromatous plaque thickness, CIMT, plaque thickness area in the extracranial carotid artery, blood pressure, HDL-C, LDL-C/HDL-C, average percentage change in LDL-C after treatment, average percentage change in TC after treatment, TG, the average percentage change in TG after treatment, VLDL-C, AI, abdominal girth, weight, BMI, waist-hip ratio, waistline, half-year recurrence rate, controlled HDL-C rate, controlled LDL-C rate, controlled TC rate, controlled TG rate, recovery rate overall response rate, change in coronary atherosclerosis, cerebrovascular events, tongue manifestation, ALT, AST, Cr, BUN, Adverse reaction, AEs, Patient-reported symptoms, cost, fatty liver
*Consensus in*	<15% scoring 1 to 3 AND ≥70% scoring 7 to 9 in each stakeholder group	cardiovascular events, LDL-C, risk of cardiovascular disease and TC

LDL-C: low density lipoprotein cholesterol, TC: total cholesterol, CIMT: carotid intima-media thickness, HDL-C: high-density lipoprotein cholesterol, TG: triglycerides, VLDL-C: very low-density lipoprotein cholesterol, AI: arteriosclerosis index, BMI: body mass index, AST: aspartate aminotransferase, ALT: alanine aminotransferase, Cr: creatinine, BUN: blood urea nitrogen, AEs: adverse events.

### 3.3 Consensus Meeting

Due to the COVID-19 outbreak and the large number of outcomes that needed a consensus, the consensus meeting was divided into two meetings. The first meeting was held on 21 August 2020, and involved 19 participants representing six stakeholders (see Table 1). The second meeting was held on 16 September 2020, and involved 12 participants representing five stakeholders (see Table 1). At the beginning of each consensus meeting, we showed the results of the three Delphi survey rounds to the participants. Outcomes of “consensus in” in the third round were voted on as either “yes” or “no” anonymously. After discussion and voting, all four “consensus in” outcomes were included in the final COS-CM-Hyperlipidemia. Outcomes of “without consensus” were discussed and re-scored using the same 9-point Likert scale.

At the end, 10 outcomes were included in the final COS-CM-Hyperlipidemia: cardiovascular events, LDL-C, risk of cardiovascular disease, TC, CIMT, HDL-C, TG, cerebrovascular events, adverse drug reaction, and patient-reported symptoms (see Table 3).

**TABLE 3:** The final COS-CM-Hyperlipidemia.

No	Outcome Name	Definition
1	Cardiovascular events	Any incidents that may cause damage to the heart
2	LDL-C	LDL-C level in the blood
3	Risk of cardiovascular disease	Typically a score, calculated by several instruments, and predicting the risk of cardiovascular disease over the next few years, for example, the QRISK model Hippisley-Cox et al. (2010)
4	TC	TC level in the blood
5	CIMT	Carotid intima-media thickness
6	HDL-C	HDL-C level in the blood
7	TG	TG level in the blood
8	Cerebrovascular events	A clinical syndrome caused by a disrupted blood supply to the brain, characterized by rapidly developing signs of focal or global disturbance of cerebral functions, lasting for more than 24 h or leading to death
9	Adverse drug reactions	In the pre-approval clinical experience with a new medicinal product or its new usages, particularly as the therapeutic dose(s) may not be established: all noxious and unintended responses to a medicinal product related to any dose should be considered adverse drug reactions^a^
10	Patient-reported symptoms	Symptoms reported by patients themselves

a International Council for Harmonisation of Technical Requirements for Pharmaceuticals for Human Use (ICH) (2021). *Integrated Addendum to ICH*. E6 (R1): *Guidelines for Good Clinical Practice*. E6 (R2): https://database.ich.org/sites/default/files/E6_R2_Addendum.pdf [Accessed 12 March 2021].

LDL-C: low density lipoprotein cholesterol, TC: total cholesterol, CIMT: carotid intima-media thickness, HDL-C: high-density lipoprotein cholesterol, TG: triglycerides.

## 4 Discussion

In this study, we identified 10 core outcomes that should be reported in all future trials involving hyperlipidemia patients receiving CM. The final COS-CM-Hyperlipidemia includes cardiovascular events, LDL-C, risk of cardiovascular disease, TC, CIMT, HDL-C, TG, cerebrovascular events, adverse drug reactions, and patient-reported symptoms. This is the first study to report the development of a COS on clinical trials of CM for hyperlipidemias.

Adoption of this COS will standardize outcome selection and reporting; this will improve the relevance and interpretability of future systematic review activities, thereby promoting evaluation of the effects of CM for hyperlipidemia. From a clinical data perspective, adoption of the COS-CM-Hyperlipidemia will contribute to the standardization of the outcome data acquisition, hence, facilitating the harmonization and standardization of source data. Thereby, it can promote clinical data sharing and merging.

When this COS is applied, three points should be noted. First, COS is a minimum outcome set, so besides outcome in COS, studies with different purposes can add other outcomes if necessary. Second, outcomes in COS do not distinguish between primary outcomes and secondary outcomes, and studies can utilize one or more outcomes in this COS as the primary outcome(s) according to their main purposes. Third, although outcome measure points are not restricted, they should be defined based on the trial objective, in terms of scientificity, rationality and feasibility (Jin et al., 2020).

There are several limitations in this study. Firstly, there is a limited geographical representation of stakeholders. Most of the experts and patients were from China, so their perspectives may not reflect those of other regions overseas. Secondly, the consensus process was divided into two parts, and some of the experts participated in the meeting via conference calls instead of face-to-face. This may have led to insufficient discussion, and thus affected the consensus results. Thirdly, we changed the outcome criteria retained for Round 3. The original criterion was that any outcomes whose median was ≥ 4 (by any stakeholder group) would continue to Delphi Round 3 (Li et al., 2019). However, when we conducted the analysis using this criterion, we found that our pre-defined criteria were too loose to eliminate outcomes. After reading the COS handbook (Williamson et al., 2017) and consulting with the SAG, we changed the criteria such that outcomes that were scored 7–9 by a majority of the panelists in Round 2 were retained for Delphi Round 3. This change may have affected the Delphi results. Although we extended Delphi Round 1’s response time and also sent personalized reminder emails or made direct telephone calls, the attrition rate in Delphi Round 1 was 30%, which exceeded the acceptable range in most situations (Williamson et al., 2017). Attrition bias may have occurred due to this lower response rate. However, in Delphi Rounds 2 and 3, the response rates were higher and acceptable. Fifthly, we did not recommend any outcome measurement instruments for this COS. This is because we knew that incomparable scores from different instruments, as well as variability in the measures’ quality (i.e., reliability and validity), would skew reporting, making it difficult to compare and combine the findings in systematic reviews and meta-analyses (Prinsen et al., 2016; Williamson et al., 2017). Therefore, we will search, evaluate, and provide generic recommendations on the selection of outcome measurement instruments for outcomes included in this COS in the future.

Next, we will collaborate with systematic review groups, clinicians, journal editors, and other stakeholders to promote the broad application of COS in hyperlipidemia. We hope this COS will be recommended by relevant industry associations. With the development of medical research, the knowledge of disease, diagnosis, treatment, and evaluation will be constantly updated, so the COS needs constant evaluation and upgrades following the latest achievements of basic and clinical research. With the constant evaluation and update, the COS keeps its value and advantage in clinical research on CM in hyperlipidemia by adding new outcomes to ensure practicability and advancement (Li et al., 2019).

This COS involved the outcomes of interventional clinical trials, not only from the perspective of disease, but also combined with the purpose of CM intervention so that the effects of CM interventions can be evaluated. Our study is similar to Qiu et al.‘s study in which they developed a COS to support CM COVID-19 treatment (Qiu et al., 2020), and Sun et al.‘s study in which they developed a COS for CM for chronic hepatitis B (Sun et al., 2020). Both of these two studies are also aimed to develop a COS for CM. what is more, the methodology and procedure of our study are similar to these two studies, too. Besides, we think this COS is appropriate for western medicine for hyperlipidemia as well, because the outcomes included in this COS are generally used. For the efficacy evaluation of CM, CM patterns have been thought as important as other general outcomes that are appropriate for both CM and western medicine (Zhang et al., 2013; Xing et al., 2014; Qiu et al., 2021). A syndrome score is the commonly used outcome for assessing the effect of CM patterns. For example, Peng et al.‘s study found that compared with western medicine, CM can significantly improve the curative effect of CM syndromes in hyperlipidemia patients with turbid phlegm syndrome (Peng et al., 2017). However, there is a lack of agreeable and universal standards for CM syndrome score, many works need to do to promote its applicability and acceptability (Luo et al., 2015). Developing a scientific, standard syndrome scale or a new more specific outcome for evaluating the effect of patterns may be one of the solutions.

In conclusion, this study has identified a COS for clinical trials of CM for hyperlipidemias. Adoption of this COS will standardize outcome selection and reporting. We also hope the use of this COS will improve the quality of (and reduce waste in) human, physical and financial resources in clinical trials of CM for hyperlipidemias. Further work is needed to explore the optimal methods for measuring these outcomes.

## Data Availability

The original contributions presented in the study are included in the article/Supplementary Material, further inquiries can be directed to the corresponding authors.

## Footnotes

1 WHO. Cardiovascular Diseases (2021). https://www.who.int/health-topics/cardiovascular-diseases#tab=tab_1 [accessed 12 March 2021]

2 Core Outcome Measures in Effectiveness Trials (2021). https://www.comet-initiative.org/[Accessed 12 March 2021]

3 Zehuai Wen, Xiankun Chen, Geng Li, et al. (2021). A core outcome set for clinical trials of Chinese Medicine in hyperlipidemia. http://www.comet-initiative.org/studies/details/983 [Accessed 12 March 2021]

## Abbreviations

CVDs, cardiovascular diseases; COS, core outcome set; CM, Chinese medicine; COS-CM-Hyperlipidemia, COS for clinical trials of Chinese Medicine for hyperlipidemias; COMET, Core Outcome Measures in Effectiveness Trials; LDL-C, low density lipoprotein cholesterol; TC, total cholesterol; CIMT, carotid intima-media thickness; HDL-C, high-density lipoprotein cholesterol; TG, triglycerides; VLDL-C, very low-density lipoprotein cholesterol; AI, arteriosclerosis index; BMI, body mass index; AST, aspartate aminotransferase; ALT, alanine aminotransferase; Cr, creatinine; BUN, blood urea nitrogen; AEs, adverse events.
