# Supplementary material for: Developing a Core Outcome Set for Clinical Trials of Chinese Medicine for Hyperlipidemia
Source: Front Pharmacol. 2022 May 2;13:847101. doi: 10.3389/fphar.2022.847101 (PMC9108338; doi:10.3389/fphar.2022.847101)
Supplement: Supplementary file 1 [file Table1.DOCX]

Table s1 outcomes and their median of each stakeholders of each Delphi round

| **No.** | **outcome** | **The 1^st^ round** | | | | | | **The 2^nd^ round** | | | | | | **The 3^rd^ round** | | | | | |
| --- | --- | --- | --- | --- | --- | --- | --- | --- | --- | --- | --- | --- | --- | --- | --- | --- | --- | --- | --- |
|  |  | **Patients** | **Clinicians or researcher in CM/integrated Chinese and Western medicine** | **Clinical pharmacy** | **Clinical epidemiology** | **Statistics** | **Editors of important relevant journals** | **Patients** | **Clinicians or researcher in CM/integrated Chinese and Western medicine** | **Clinical pharmacy** | **Clinical epidemiology** | **Statistics** | **Editors of important relevant journals** | **Patients** | **Clinicians or researcher in CM/integrated Chinese and Western medicine** | **Clinical pharmacy** | **Clinical epidemiology** | **Statistics** | **Editors of important relevant journals** |
| 1 | All-cause mortality # | 5 | 5 | 7.5 | 8 | 3.5 | 5.5 | 7.5 | 5 | 7 | 9 | 4 | 2.5 |  |  |  |  |  |  |
| 2 | Number of deaths # | 6 | 4 | 5 | 3 | 3.5 | 2.5 | 7 | 4 | 6 | 3 | 3.5 | 2.5 |  |  |  |  |  |  |
| 3 | APO-A # | 6 | 5 | 5 | 4 | 7.5 | 6 | 6 | 5 | 5.5 | 4 | 7 | 5 |  |  |  |  |  |  |
| 4 | APO-A/B # | 6 | 5 | 4 | 4 | 7.5 | 5.5 | 6 | 5 | 5 | 4 | 7 | 5.5 |  |  |  |  |  |  |
| 5 | APO-AI # | 6 | 5 | 4 | 4 | 8 | 5 | 6 | 5 | 4 | 4 | 7 | 4.5 |  |  |  |  |  |  |
| 6 | APO-AII # | 6 | 5 | 4 | 4 | 8 | 5 | 6 | 5 | 4 | 4 | 7 | 4.5 |  |  |  |  |  |  |
| 7 | APO-B # | 6 | 5 | 5.5 | 4 | 7.5 | 5.5 | 6 | 5 | 5.5 | 4 | 7 | 5 |  |  |  |  |  |  |
| 8 | Sebum thickness # | 8 | 5 | 5 | 4.5 | 7.5 | 4 | 8 | 6 | 5 | 4.5 | 7.5 | 4.5 |  |  |  |  |  |  |
| 9 | percentage of body fat # | 7 | 6 | 6 | 5.5 | 6.5 | 5.5 | 8 | 6 | 6 | 5 | 6.5 | 5.5 |  |  |  |  |  |  |
| 10 | Plaque size | 7 | 7 | 6 | 7 | 5.5 | 6 | 7 | 8 | 6 | 7 | 6.5 | 6 | 7 | 7 | 6.5 | 7 | 6.5 | 7 |
| 11 | Plaque area | 7 | 7 | 6 | 7 | 5.5 | 6 | 7 | 7 | 6 | 7 | 6.5 | 6 | 7 | 7 | 6.5 | 7 | 6.5 | 6 |
| 12 | Number of plaques | 7 | 7 | 5.5 | 6.5 | 5.5 | 6 | 7 | 7 | 6 | 6.5 | 6 | 6 | 7.5 | 7 | 7 | 7 | 6.5 | 6.5 |
| 13 | Thickness of atheromatous plaque | 7 | 7 | 7 | 8 | 5.5 | 6.5 | 7.5 | 7 | 7 | 8 | 6.5 | 6.5 | 7.5 | 7 | 7 | 7.5 | 7 | 6.5 |
| 14 | IMT | 6 | 7 | 7 | 7.5 | 5.5 | 7 | 6.5 | 7 | 7 | 7.5 | 6.5 | 7 | 7.5 | 7 | 7 | 7 | 7 | 7 |
| 15 | Area of plaque thickness in extracranial carotid artery | 6 | 7 | 7 | 5.5 | 5.5 | 7 | 6.5 | 7 | 7 | 7 | 6 | 7 | 6 | 7 | 7 | 7 | 6.5 | 7 |
| 16 | electrocardiogram # | 7 | 5 | 6 | 2.5 | 4.5 | 5 | 7 | 5 | 6 | 3 | 4.5 | 2.5 |  |  |  |  |  |  |
| 17 | Fasting blood glucose # | 7 | 6 | 5.5 | 5 | 5 | 4.5 | 7.5 | 6 | 6 | 5 | 5 | 4 |  |  |  |  |  |  |
| 18 | HbA1c # | 7 | 6 | 6.5 | 5 | 5 | 4.5 | 6 | 6 | 6 | 5 | 5 | 4.5 |  |  |  |  |  |  |
| 19 | Blood pressure | 8 | 7 | 7 | 5.5 | 5 | 4.5 | 8 | 7 | 7 | 5 | 7 | 4 | 8 | 7 | 7.5 | 5.5 | 7 | 4 |
| 20 | HDL-C | 8 | 7 | 7.5 | 6.5 | 5.5 | 7 | 8 | 7 | 7.5 | 6.5 | 6 | 8.5 | 8 | 7 | 7 | 7 | 7 | 8.5 |
| 21 | LDL-C | 8 | 8 | 8 | 7 | 7 | 7 | 8 | 8 | 8 | 8 | 8 | 8.5 | 8 | 8 | 8.5 | 8 | 8.5 | 8.5 |
| 22 | LDL-C/HDL-C | 8 | 6 | 7 | 7 | 6.5 | 7 | 7.5 | 7 | 8 | 7.5 | 7.5 | 8.5 | 7.5 | 6 | 8 | 7.5 | 7.5 | 8 |
| 23 | The average percentage change of LDL-C after treatment | 8 | 7 | 8 | 5 | 7.5 | 6.5 | 8 | 7 | 8 | 7 | 8 | 8.5 | 8 | 7 | 8 | 7 | 8 | 8.5 |
| 24 | TC | 8 | 7 | 6.5 | 6.5 | 7 | 7 | 8 | 7 | 6.5 | 7 | 7.5 | 8 | 8 | 7 | 7 | 7 | 7.5 | 8 |
| 25 | The average percentage change of TC after treatment | 8 | 6 | 7 | 6.5 | 8 | 7 | 8 | 7 | 7 | 7 | 7.5 | 8.5 | 7.5 | 7 | 7 | 7 | 7 | 7.5 |
| 26 | TG | 8 | 7 | 6 | 6.5 | 7 | 7 | 8 | 7 | 6 | 6.5 | 7 | 8 | 8 | 7 | 6.5 | 7 | 6.5 | 8 |
| 27 | The average percentage change of TG after treatment | 7 | 6 | 6.5 | 5.5 | 8.5 | 8 | 7.5 | 7 | 6.5 | 7.5 | 7.5 | 8.5 | 8 | 7 | 6.5 | 7 | 8 | 7.5 |
| 28 | VLDL-C | 7 | 7 | 6 | 6 | 5.5 | 6 | 6.5 | 7 | 6 | 7 | 5.5 | 7.5 | 6.5 | 7 | 6 | 7 | 5 | 7.5 |
| 29 | AI | 7 | 7 | 7.5 | 6 | 6.5 | 7 | 7.5 | 7 | 7.5 | 6 | 7 | 7.5 | 8 | 7 | 7 | 6.5 | 7 | 7.5 |
| 30 | Non-HDL-C # | 7 | 6 | 8 | 5.5 | 5.5 | 5.5 | 7.5 | 6 | 8 | 6 | 5.5 | 7 |  |  |  |  |  |  |
| 31 | Abdominal girth | 7 | 6 | 7 | 4.5 | 5 | 6 | 7 | 6 | 7 | 6 | 5 | 5 | 7 | 7 | 7 | 6 | 6 | 6.5 |
| 32 | weight | 7 | 6 | 5 | 5 | 5 | 4.5 | 7 | 7 | 5 | 6 | 5 | 4.5 | 7 | 7 | 6 | 6 | 5.5 | 5 |
| 33 | BMI | 7 | 7 | 5.5 | 5.5 | 5.5 | 6 | 7.5 | 7 | 5.5 | 6 | 5.5 | 6 | 8 | 7 | 6 | 6 | 5.5 | 6 |
| 34 | Hipline # | 6 | 6 | 6.5 | 4.5 | 5 | 5 | 6.5 | 7 | 6.5 | 6 | 5 | 5 |  |  |  |  |  |  |
| 35 | waist-hip ratio | 7 | 6 | 7 | 5.5 | 5.5 | 7 | 7 | 7 | 7 | 6 | 6 | 7 | 7.5 | 7 | 7 | 6.5 | 6.5 | 7 |
| 36 | waistline | 7 | 6 | 7 | 4.5 | 5 | 5.5 | 7 | 7 | 7 | 6 | 5 | 5.5 | 7 | 6 | 7 | 6 | 5.5 | 6 |
| 37 | Half-year recurrence rate | 8 | 7 | 8 | 7 | 6 | 6.5 | 6.5 | 7 | 8 | 7 | 6.5 | 6 | 6 | 7 | 7 | 6.5 | 7 | 6 |
| 38 | Controlled rate of HDL-C | 8 | 6 | 6.5 | 6.5 | 7 | 6 | 8 | 6 | 6.5 | 6.5 | 7.5 | 6 | 7 | 6 | 6.5 | 7 | 7 | 6 |
| 39 | Controlled rate of LDL-C | 8 | 7 | 7 | 7 | 8.5 | 6 | 7.5 | 8 | 7 | 7 | 8.5 | 6 | 7.5 | 8 | 7.5 | 7.5 | 7.5 | 7 |
| 40 | Controlled rate of TC | 8 | 6 | 7 | 6.5 | 8 | 6 | 8 | 7 | 7 | 6.5 | 7.5 | 6 | 7.5 | 7 | 7 | 7 | 8 | 6.5 |
| 41 | Controlled rate of TG | 8 | 6 | 6.5 | 6.5 | 8 | 6 | 7.5 | 7 | 7 | 6.5 | 8 | 6 | 7.5 | 7 | 7 | 7 | 8 | 6.5 |
| 42 | Recovery rate | 7 | 6 | 6.5 | 7 | 6.5 | 6 | 7 | 7 | 6.5 | 7 | 7.5 | 6 | 7.5 | 7 | 7 | 7 | 7 | 6 |
| 43 | Overall response rate | 8 | 6 | 7.5 | 6.5 | 8.5 | 7 | 7.5 | 6 | 7.5 | 6.5 | 9 | 6.5 | 8 | 6 | 7.5 | 7 | 8 | 6.5 |
| 44 | change of coronary atherosclerosis | 7 | 7 | 8 | 6 | 6 | 6.5 | 7 | 8 | 8.5 | 7 | 6.5 | 7 | 7 | 8 | 8.5 | 7.5 | 8 | 7 |
| 45 | Risk of cardiovascular disease | 7 | 7 | 8 | 7 | 6.5 | 7 | 8 | 8 | 8 | 7.5 | 7 | 7 | 7.5 | 8 | 8 | 7.5 | 7 | 7 |
| 46 | Cardiovascular events | 8 | 8 | 8.5 | 8.5 | 6.5 | 7.5 | 8 | 8 | 8.5 | 8 | 7.5 | 7.5 | 8 | 9 | 9 | 8 | 7.5 | 7.5 |
| 47 | Cerebrovascular events * |  |  |  |  |  |  | 7 | 8 | 7 | 8 | 7 | 6.5 | 7.5 | 8 | 7.5 | 8 | 7.5 | 7 |
| 48 | Pulse condition # | 7 | 6 | 6.5 | 4.5 | 5 | 4.5 | 7.5 | 6 | 7 | 5 | 6.5 | 4.5 |  |  |  |  |  |  |
| 49 | tongue manifestation | 7 | 6 | 6.5 | 4.5 | 5.5 | 4.5 | 8 | 6 | 6.5 | 5 | 6 | 4.5 | 7.5 | 7 | 7 | 5.5 | 5 | 4.5 |
| 50 | constitution score # | 6 | 6 | 5.5 | 5.5 | 4 | 4.5 | 6.5 | 6 | 6 | 5 | 4 | 5 |  |  |  |  |  |  |
| 51 | the grade of CM syndrome  # | 7 | 5 | 5.5 | 5 | 4.5 | 5 | 7 | 6 | 5.5 | 5 | 5 | 5 |  |  |  |  |  |  |
| 52 | The score of CM syndrome (Secondary symptoms) # | 6 | 6 | 5.5 | 5 | 4.5 | 4.5 | 7 | 6 | 5.5 | 5 | 5.5 | 5 |  |  |  |  |  |  |
| 53 | The score of CM syndrome (primary symptoms) # | 6 | 6 | 5.5 | 5.5 | 5.5 | 5.5 | 7 | 6 | 5.5 | 5.5 | 6 | 5.5 |  |  |  |  |  |  |
| 54 | The efficacy on CM syndrome (Single item) # | 6 | 6 | 6.5 | 5.5 | 5 | 3.5 | 7 | 5 | 6.5 | 5 | 4.5 | 4 |  |  |  |  |  |  |
| 55 | The efficacy on CM syndrome (entirety) # | 6 | 6 | 6.5 | 5.5 | 5 | 5 | 7 | 6 | 6.5 | 6 | 6.5 | 5 |  |  |  |  |  |  |
| 56 | Disappearance rate of CM syndromes # | 6 | 6 | 7 | 5.5 | 3.5 | 4.5 | 7 | 6 | 7 | 5.5 | 4.5 | 4 |  |  |  |  |  |  |
| 57 | GGT # | 8 | 5 | 4.5 | 5.5 | 6.5 | 5.5 | 7 | 6 | 4.5 | 5 | 7.5 | 5 |  |  |  |  |  |  |
| 58 | ALT | 8 | 6 | 6.5 | 6.5 | 6.5 | 6 | 7 | 7 | 6.5 | 5 | 7.5 | 5.5 | 7 | 7 | 7 | 6 | 7 | 5.5 |
| 59 | AST | 8 | 6 | 6.5 | 5.5 | 6.5 | 5.5 | 7 | 7 | 6.5 | 5 | 7.5 | 5.5 | 7 | 7 | 7 | 5.5 | 7 | 5.5 |
| 60 | TP # | 8 | 5 | 4.5 | 5.5 | 5.5 | 5.5 | 7 | 5 | 4.5 | 5 | 6 | 4.5 |  |  |  |  |  |  |
| 61 | Cr | 8 | 7 | 6.5 | 6 | 6.5 | 6 | 7 | 7 | 6.5 | 5 | 7.5 | 5.5 | 7 | 7 | 7 | 6 | 7.5 | 5.5 |
| 62 | BUN | 8 | 6 | 6.5 | 6 | 6.5 | 5 | 7 | 6 | 6.5 | 5 | 7.5 | 5.5 | 7 | 7 | 7 | 6 | 7 | 5.5 |
| 63 | UA # | 8 | 6 | 6 | 5 | 6.5 | 5 | 7.5 | 6 | 6 | 4.5 | 6.5 | 5.5 |  |  |  |  |  |  |
| 64 | Adverse reaction | 8 | 6 | 7 | 7 | 7.5 | 4.5 | 8 | 7 | 7 | 7 | 8 | 5.5 | 7.5 | 7 | 7 | 7 | 7 | 5.5 |
| 65 | AEs | 8 | 6 | 6 | 6 | 8 | 4 | 7.5 | 6 | 6 | 6 | 8 | 4.5 | 8 | 7 | 6 | 6.5 | 7.5 | 4.5 |
| 66 | Patients reported symptoms | 7 | 6 | 4 | 5.5 | 7 | 5 | 8 | 7 | 3.5 | 5.5 | 7 | 5 | 8 | 7 | 5 | 5.5 | 7 | 5 |
| 67 | Daily activity # | 7 | 6 | 5 | 3 | 5.5 | 4 | 8 | 7 | 5 | 4 | 6.5 | 5 |  |  |  |  |  |  |
| 68 | Daily energy consuming # | 7 | 7 | 5.5 | 3 | 5.5 | 4 | 7.5 | 8 | 5.5 | 4 | 5.5 | 5 |  |  |  |  |  |  |
| 69 | Quality of life # | 7 | 6 | 5.5 | 5.5 | 7 | 6.5 | 7 | 6 | 6 | 6 | 7 | 6 |  |  |  |  |  |  |
| 70 | Score of health survey # | 7 | 6 | 5.5 | 5 | 6 | 5 | 7 | 5 | 5.5 | 5 | 5.5 | 4.5 |  |  |  |  |  |  |
| 71 | Items of health and pleasure | 7 | 6 | 5.5 | 5 | 6 | 4.5 | 7 | 5 | 5.5 | 5 | 7.5 | 4.5 |  |  |  |  |  |  |
| 72 | Cost | 7 | 6 | 7 | 4.5 | 5.5 | 4.5 | 8 | 6 | 7 | 5 | 6 | 5 | 8 | 6 | 7 | 6 | 5.5 | 5 |
| 73 | fatty liver * |  |  |  |  |  |  | 8 | 7 | 6 | 5.5 | 7 | 6 | 8 | 7 | 7.5 | 7 | 7.5 | 6 |

* New outcome that suggested by panelists in the first round

# Deleted outcomes in the second round

APO-A: Apolipoproteins -A, APO-A/B: ApolipoproteinsA/B, APO-AI: Apolipoproteins -AI, APO-AII: Apolipoproteins -AII, APO-B: Apolipoproteins -B, IMT: carotid intima-media thickness, HDL-C: High-density lipoprotein Cholesterol, LDL-C: Low Density Lipoprotein Cholesterol, TC: Total Cholesterol, TG: triglyceride, VLDL-C: Very Low Density Lipoprotein Cholesterol, AI: Arteriosclerosis index, CM: Chinese medicine, BMI: Body mass index, GGT: Gamma-Glutamyl Transpeptidase, AST: Aspartate Aminotransferase, ALT: Alanine Aminotransferase, TP: total protein, Cr: Creatinine, BUN: Blood urea nitrogen, UA: Uric acid, AEs: Adverse events.

Table s2 outcomes that suggested by panelists in Delphi round 1

| No. | Outcome name | Included in Delphi round 2? (Y/N) |
| --- | --- | --- |
| 1 | Fatty liver | Y |
| 2 | Menopause | N |
| 3 | Smoking | N |
| 4 | Location of plaque | N |
| 5 | MR on carotid artery | N |
| 6 | Cerebrovascular events | Y |
