# Supplementary figures and images for: Developing a Core Outcome Set for Clinical Trials of Chinese Medicine for Hyperlipidemia
Source: Front Pharmacol. 2022 May 2;13:847101. doi: 10.3389/fphar.2022.847101 (PMC9108338; doi:10.3389/fphar.2022.847101)

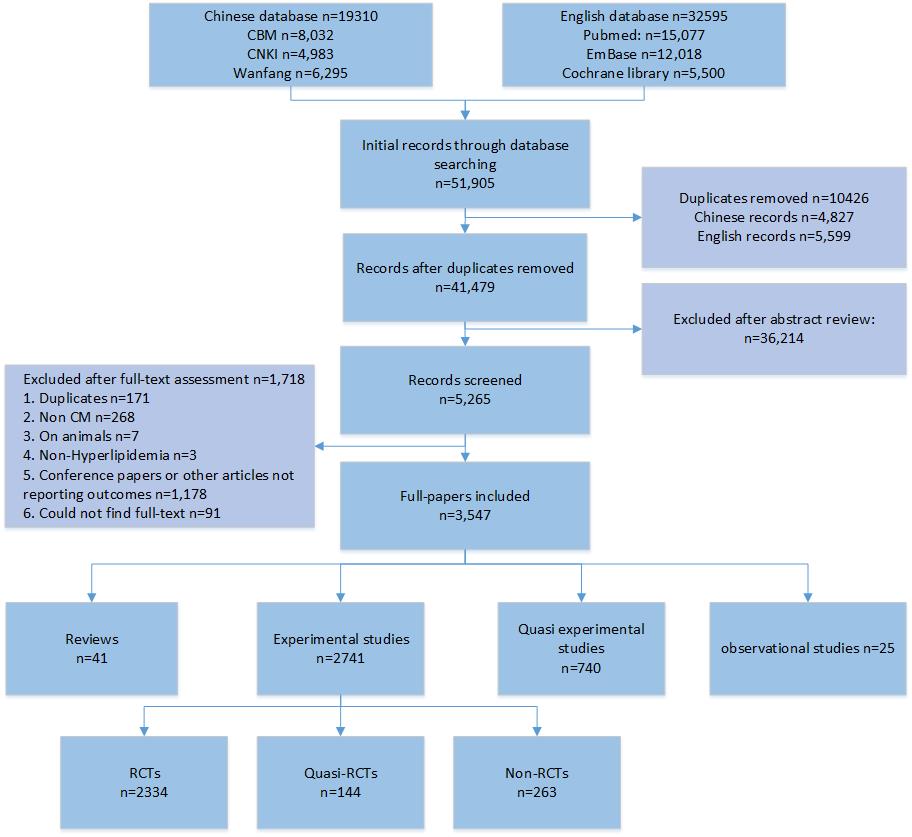

Supplement: Supplementary file 2 [file Image1.JPEG]
