# Supplementary material for: Developing a Core Outcome Set for Clinical Trials of Chinese Medicine for Hyperlipidemia
Source: Front Pharmacol. 2022 May 2;13:847101. doi: 10.3389/fphar.2022.847101 (PMC9108338; doi:10.3389/fphar.2022.847101)
Supplement: Supplementary file 3 [file Table2.docx]

| **ID** | **Outcome name** | **NO. of** **studies involved** | **Reported as primary or secondary outcome** | | | **Definition** | |
| --- | --- | --- | --- | --- | --- | --- | --- |
|  |  |  | **NO. of studies Reported as Secondary outcome** | **NO. of studies Reported as primary outcome** | **NO. of studies did not mention** | **NO. of studies without definition** | **NO. of studies with definition** |
| 1 | liver function | 1199 | 6 | 1 | 1192 | 1189 | 10 |
| 2 | renal function | 1046 | 6 | 1 | 1039 | 1036 | 10 |
| 3 | blood routine examination | 958 | 5 | 1 | 952 | 949 | 9 |
| 4 | routine urine examination | 873 | 5 | 1 | 867 | 866 | 7 |
| 5 | stool routine examination | 376 | 5 | 1 | 370 | 370 | 6 |
| 6 | glutamic-pyruvic transaminase (ALT) | 292 | 4 | 2 | 286 | 292 | 0 |
| 7 | urea nitrogen (BUN) | 239 | 0 | 0 | 239 | 239 | 0 |
| 8 | Creatinine (Cr) | 233 | 2 | 0 | 231 | 232 | 1 |
| 9 | Hematocrit (HCT) | 181 | 1 | 2 | 178 | 179 | 2 |
| 10 | glutamic oxalacetic transaminase (AST) | 152 | 2 | 1 | 149 | 152 | 0 |
| 11 | erythrocyte sedimentation rate (ESR) | 64 | 0 | 0 | 64 | 63 | 1 |
| 12 | Uric Acid (UA) | 54 | 0 | 1 | 53 | 54 | 0 |
| 13 | Platelets (PLT) | 45 | 0 | 0 | 45 | 45 | 0 |
| 14 | white blood cell (WBC) | 25 | 0 | 0 | 25 | 25 | 0 |
| 15 | total bilirubin (TBIL) | 24 | 0 | 0 | 24 | 24 | 0 |
| 16 | glutamyl transpeptidase (GGT) | 19 | 0 | 0 | 19 | 19 | 0 |
| 17 | red blood cell (RBC) | 19 | 0 | 0 | 19 | 19 | 0 |
| 18 | alkaline phosphatase (AKP) | 17 | 0 | 0 | 17 | 17 | 0 |
| 19 | hemoglobin (Hb) | 16 | 0 | 0 | 16 | 16 | 0 |
| 20 | serum albumin (ALB) | 13 | 0 | 0 | 13 | 13 | 0 |
| 21 | total serum protein (TP) | 12 | 0 | 0 | 12 | 12 | 0 |
| 22 | urine glucose | 11 | 0 | 0 | 11 | 11 | 0 |
| 23 | K equation of erythrocyte sedimentation rate | 10 | 0 | 0 | 10 | 10 | 0 |
| 24 | liver enzyme | 9 | 1 | 0 | 8 | 9 | 0 |
| 25 | urine protein (PRO) | 9 | 0 | 0 | 9 | 9 | 0 |
| 26 | Stool Occult Blood | 6 | 0 | 1 | 5 | 6 | 0 |
| 27 | total protein | 6 | 0 | 0 | 6 | 6 | 0 |
| 28 | occult blood test | 6 | 0 | 0 | 6 | 6 | 0 |
| 29 | urine occult blood (BLD) | 4 | 0 | 0 | 4 | 4 | 0 |
| 30 | classification of peripheral white blood cells | 4 | 0 | 0 | 4 | 4 | 0 |
| 31 | total peripheral white blood cells | 4 | 0 | 0 | 4 | 4 | 0 |
| 32 | hemobilirubin | 4 | 0 | 0 | 4 | 4 | 0 |
| 33 | K equation of erythrocyte sedimentation | 2 | 0 | 0 | 2 | 2 | 0 |
| 34 | urine ketone(ket) | 2 | 0 | 0 | 2 | 2 | 0 |
| 35 | PH | 2 | 0 | 0 | 2 | 2 | 0 |
| 36 | direct bilirubin (DBIL) | 3 | 0 | 0 | 3 | 3 | 0 |
| 37 | Neutral lymphocyte | 2 | 0 | 0 | 2 | 2 | 0 |
| 38 | mean corpuscular volume | 1 | 0 | 0 | 1 | 1 | 0 |
| 39 | lymphocyte | 1 | 0 | 0 | 1 | 1 | 0 |
| 40 | Urine microalbuminuria | 1 | 0 | 0 | 1 | 1 | 0 |
| 41 | mean platelet volume | 1 | 0 | 0 | 1 | 1 | 0 |
| 42 | prealbumin | 1 | 0 | 0 | 1 | 1 | 0 |
| 43 | K equation of blood flow | 1 | 0 | 0 | 1 | 1 | 0 |
| 44 | hemanalysis and urinalysis | 1 | 0 | 0 | 1 | 1 | 0 |
| 45 | Mean blood cell volume | 1 | 0 | 0 | 1 | 1 | 0 |
| 46 | neutrophile granulocyte | 1 | 0 | 0 | 1 | 1 | 0 |
| 47 | Aspartic acid | 1 | 0 | 0 | 1 | 1 | 0 |
| 48 | cytokine level | 1 | 0 | 0 | 1 | 1 | 0 |
| 49 | Paraoxonase-1 | 1 | 0 | 0 | 1 | 1 | 0 |
| 50 | Total antioxidant state | 2 | 0 | 0 | 2 | 2 | 0 |
| 51 | Total oxidation state | 2 | 0 | 0 | 2 | 2 | 0 |
| 52 | rate of adverse reaction | 10 | 0 | 0 | 10 | 4 | 6 |
| 53 | adverse reaction | 9 | 0 | 1 | 8 | 4 | 5 |
| 54 | total rate of adverse reaction | 1 | 0 | 0 | 1 | 1 | 0 |
| 55 | adverse event | 85 | 3 | 1 | 81 | 85 | 0 |
| 56 | rate of adverse event | 1 | 0 | 0 | 1 | 1 | 0 |
| 57 | total effective rate | 2780 | 6 | 18 | 2756 | 132 | 2648 |
| 58 | improved rate | 16 | 0 | 0 | 16 | 5 | 11 |
| 59 | control rate in TC | 4 | 0 | 0 | 4 | 2 | 2 |
| 60 | relationship between CM syndrome and efficacy | 4 | 0 | 0 | 4 | 4 | 0 |
| 61 | achieved rate | 4 | 0 | 0 | 4 | 0 | 4 |
| 62 | control rate in LDL-C | 3 | 0 | 0 | 3 | 2 | 1 |
| 63 | control rate in TG | 3 | 0 | 0 | 3 | 1 | 2 |
| 64 | recovery rate | 2 | 0 | 0 | 2 | 1 | 1 |
| 65 | recurrence rate | 2 | 0 | 0 | 2 | 0 | 2 |
| 66 | control rate | 2 | 0 | 0 | 2 | 0 | 2 |
| 67 | cured rate | 3 | 0 | 0 | 3 | 1 | 2 |
| 68 | Half-year recurrence rate | 1 | 0 | 0 | 1 | 1 | 0 |
| 69 | relationship between course of disease and efficacy | 1 | 0 | 0 | 1 | 1 | 0 |
| 70 | relationship between age and efficacy | 1 | 0 | 0 | 1 | 1 | 0 |
| 71 | relationship between treatment period and efficacy | 1 | 0 | 0 | 1 | 1 | 0 |
| 72 | control rate in HDL-C | 1 | 0 | 0 | 1 | 1 | 0 |
| 73 | percent of patient who reached the treatment destination | 1 | 0 | 1 | 0 | 1 | 0 |
| 74 | frequency of positive point of auricular acupoints | 1 | 0 | 0 | 1 | 1 | 0 |
| 75 | relationship between category of hyperlipidemia and efficacy | 1 | 0 | 0 | 1 | 1 | 0 |
| 76 | effective rate of hemorheology | 1 | 0 | 0 | 1 | 0 | 1 |
| 77 | rate of change of blood lipid parameters | 1 | 0 | 0 | 1 | 0 | 1 |
| 78 | percentage of change of blood lipid | 1 | 0 | 0 | 1 | 1 | 0 |
| 79 | Percentage of decline of blood lipid | 1 | 0 | 0 | 1 | 1 | 0 |
| 80 | symptoms reported by patients themselves | 1 | 0 | 0 | 1 | 1 | 0 |
| 81 | mean daily activity | 1 | 0 | 0 | 1 | 1 | 0 |
| 82 | Mean daily caloric intake | 1 | 0 | 0 | 1 | 1 | 0 |
| 83 | Dietary intake | 1 | 0 | 0 | 1 | 1 | 0 |
| 84 | quality of life | 9 | 4 | 1 | 4 | 8 | 1 |
| 85 | score of health survey | 1 | 1 | 0 | 0 | 1 | 0 |
| 86 | items of health and happiness | 1 | 0 | 0 | 1 | 1 | 0 |
| 87 | psychological test | 1 | 0 | 0 | 1 | 1 | 0 |
| 88 | Score of satisfaction by community patients | 1 | 0 | 0 | 1 | 0 | 1 |
| 89 | Total satisfaction | 2 | 0 | 0 | 2 | 2 | 0 |
| 90 | NO2/NO3 | 1 | 0 | 0 | 1 | 1 | 0 |
| 91 | apolipoprotein-B(APO-B) | 254 | 6 | 10 | 238 | 251 | 3 |
| 92 | apolipoprotein -AI(APO-AI) | 177 | 4 | 7 | 166 | 174 | 3 |
| 93 | apolipoprotein -A(APO-A) | 131 | 3 | 4 | 124 | 129 | 2 |
| 94 | α- lipoprotein | 69 | 2 | 4 | 63 | 68 | 1 |
| 95 | β- lipoprotein | 62 | 0 | 0 | 62 | 62 | 0 |
| 96 | apolipoprotein -B100(APO-B100) | 61 | 1 | 2 | 58 | 59 | 2 |
| 97 | apolipoprotein A/B(APO-A/B) | 12 | 0 | 0 | 12 | 12 | 0 |
| 98 | apolipoprotein (APO) | 11 | 0 | 2 | 9 | 11 | 0 |
| 99 | apolipoprotein AI/B (APO-AI/B) | 7 | 0 | 0 | 7 | 7 | 0 |
| 100 | homocysteine (HCY) | 8 | 0 | 0 | 8 | 7 | 1 |
| 101 | apolipoproteinAI/B100(APO-AI/B100) | 4 | 0 | 0 | 4 | 4 | 0 |
| 102 | von Willebrand factor | 3 | 0 | 0 | 3 | 3 | 0 |
| 103 | apolipoprotein-AII(APO-AII) | 3 | 0 | 0 | 3 | 3 | 0 |
| 104 | apolipoprotein-E(APO-E) | 3 | 0 | 0 | 3 | 3 | 0 |
| 105 | Cholesterol ester transfer protein | 2 | 0 | 0 | 2 | 2 | 0 |
| 106 | IgG | 2 | 0 | 0 | 2 | 2 | 0 |
| 107 | proprotein convertase subtilisin/kexin type 9, PCSK9 | 2 | 0 | 0 | 2 | 2 | 0 |
| 108 | apolipoproteinB/AI(APO-B/AI) | 2 | 0 | 0 | 2 | 2 | 0 |
| 109 | apolipoprotein-CII(APO-CII) | 2 | 0 | 0 | 2 | 2 | 0 |
| 110 | apolipoprotein-CIII(APO-CIII) | 2 | 0 | 0 | 2 | 2 | 0 |
| 111 | ALB/GLB | 1 | 0 | 0 | 1 | 1 | 0 |
| 112 | liver fatty acid binding protein(L-FABP) | 1 | 0 | 0 | 1 | 1 | 0 |
| 113 | High mobility group boxB1 | 1 | 0 | 0 | 1 | 1 | 0 |
| 114 | IgA | 1 | 0 | 0 | 1 | 1 | 0 |
| 115 | IgM | 1 | 0 | 0 | 1 | 1 | 0 |
| 116 | pre-beta-lipoprotein | 1 | 0 | 0 | 1 | 1 | 0 |
| 117 | pre-beta-lipoprotein1 | 1 | 0 | 0 | 1 | 1 | 0 |
| 118 | pre-beta-lipoprotein2 | 1 | 0 | 0 | 1 | 1 | 0 |
| 119 | Globulin (GLO) | 1 | 0 | 0 | 1 | 1 | 0 |
| 120 | chylomicron | 1 | 0 | 0 | 1 | 1 | 0 |
| 121 | Plasma Von Willebrand Factor | 1 | 0 | 0 | 1 | 1 | 0 |
| 122 | serum albumin lipid complex (AL) | 1 | 0 | 0 | 1 | 1 | 0 |
| 123 | apolipoprotein-CI(APO-CI) | 1 | 0 | 0 | 1 | 1 | 0 |
| 124 | adipocyte-type fatty acid binding protein(A-FABP) | 1 | 0 | 0 | 1 | 1 | 0 |
| 125 | Total hydroxyproline (HYP) | 1 | 0 | 0 | 1 | 1 | 0 |
| 126 | High sensitivity C-reactive protein | 5 | 0 | 0 | 5 | 5 | 0 |
| 127 | Protein cholesterol | 1 | 0 | 0 | 1 | 0 | 1 |
| 128 | tissue inhibitor of metalloproteinases-1 | 1 | 0 | 0 | 1 | 1 | 0 |
| 129 | Interferon-γ | 1 | 0 | 0 | 1 | 1 | 0 |
| 130 | γ-glutamyl transpeptidase | 1 | 0 | 0 | 1 | 1 | 0 |
| 131 | indirect bilirubin | 1 | 0 | 0 | 1 | 1 | 0 |
| 132 | Thioredoxin interaction protein | 1 | 0 | 0 | 1 | 1 | 0 |
| 133 | nesfatin-1 | 2 | 0 | 0 | 2 | 2 | 0 |
| 134 | electrolyte | 60 | 0 | 0 | 60 | 59 | 1 |
| 135 | potassium (K) | 12 | 0 | 0 | 12 | 12 | 0 |
| 136 | chlorine | 10 | 0 | 0 | 10 | 10 | 0 |
| 137 | sodium (Na) | 8 | 0 | 0 | 8 | 8 | 0 |
| 138 | calcium (Ca) | 3 | 0 | 0 | 3 | 3 | 0 |
| 139 | zinc (Zn) | 3 | 0 | 0 | 3 | 3 | 0 |
| 140 | Ferrum (Fe) | 2 | 0 | 0 | 2 | 2 | 0 |
| 141 | magnesium (Mg) | 1 | 0 | 0 | 1 | 1 | 0 |
| 142 | obesity index | 4 | 0 | 0 | 4 | 1 | 3 |
| 143 | thickness of sebum | 2 | 0 | 0 | 2 | 2 | 0 |
| 144 | water change of fat and muscle | 2 | 0 | 0 | 2 | 2 | 0 |
| 145 | content of body fat | 3 | 0 | 0 | 3 | 2 | 1 |
| 146 | fat thickness | 2 | 0 | 0 | 2 | 2 | 0 |
| 147 | Plasma obese protein (OP) | 1 | 0 | 0 | 1 | 1 | 0 |
| 148 | percentage of fat | 1 | 0 | 0 | 1 | 1 | 0 |
| 149 | microviscosity(η) | 1 | 0 | 0 | 1 | 1 | 0 |
| 150 | Degree of fluorescence polarization(P) | 1 | 0 | 0 | 1 | 1 | 0 |
| 151 | carotid intima-media thickness (IMT) | 13 | 0 | 1 | 12 | 12 | 1 |
| 152 | carotid color ultrasonography | 9 | 0 | 0 | 9 | 8 | 1 |
| 153 | carotid plaque | 3 | 0 | 0 | 3 | 3 | 0 |
| 154 | Plaque area | 2 | 0 | 0 | 2 | 2 | 0 |
| 155 | carotid color ultrasonography (plaque size) | 2 | 0 | 0 | 2 | 2 | 0 |
| 156 | plaque thickness of carotid atherosclerotic | 1 | 0 | 0 | 1 | 1 | 0 |
| 157 | carotid color ultrasonography (plaque amount) | 1 | 0 | 0 | 1 | 1 | 0 |
| 158 | 2,4dehydro-cholestanol | 1 | 0 | 0 | 1 | 1 | 0 |
| 159 | 7-alkene cholestanol | 1 | 0 | 0 | 1 | 1 | 0 |
| 160 | campesterol | 1 | 0 | 0 | 1 | 1 | 0 |
| 161 | stigmasterol | 1 | 0 | 0 | 1 | 1 | 0 |
| 162 | sitoesterol | 1 | 0 | 0 | 1 | 1 | 0 |
| 163 | squalene | 1 | 0 | 0 | 1 | 1 | 0 |
| 164 | superoxide dismutase (SOD) | 49 | 1 | 1 | 47 | 47 | 2 |
| 165 | glutathion peroxidase (GSH-px) | 6 | 0 | 1 | 5 | 6 | 0 |
| 166 | lecithin-cholesterol acyltransferase | 2 | 0 | 0 | 2 | 1 | 1 |
| 167 | lipoprotein lipase activity | 2 | 0 | 0 | 2 | 1 | 1 |
| 168 | Lecithin cholesterol acylase | 1 | 0 | 0 | 1 | 1 | 0 |
| 169 | Manganese- superoxide dismutase (Mn-SOD) | 1 | 0 | 0 | 1 | 1 | 0 |
| 170 | catalase activity | 1 | 0 | 0 | 1 | 1 | 0 |
| 171 | Copper- superoxide dismutase (Cu-SOD) | 1 | 0 | 0 | 1 | 1 | 0 |
| 172 | Zinc- superoxide dismutase (Zn-SOD) | 1 | 0 | 0 | 1 | 1 | 0 |
| 173 | serum enzyme | 1 | 0 | 0 | 1 | 1 | 0 |
| 174 | Measurement of enzymes of lipoprotein metabolism | 1 | 0 | 0 | 1 | 1 | 0 |
| 175 | Interleukin-1β | 2 | 0 | 0 | 2 | 2 | 0 |
| 176 | Endothelin | 4 | 0 | 0 | 4 | 4 | 0 |
| 177 | Eosinophil cationic protein | 3 | 0 | 0 | 3 | 3 | 0 |
| 178 | prothrombin time (PT) | 26 | 0 | 0 | 26 | 26 | 0 |
| 179 | bleeding time | 12 | 0 | 0 | 12 | 12 | 0 |
| 180 | D-dimer | 7 | 0 | 0 | 7 | 7 | 0 |
| 181 | Activated partial thromboplastin time (APTT) | 7 | 0 | 0 | 7 | 7 | 0 |
| 182 | blood coagulation factor | 5 | 0 | 0 | 5 | 5 | 0 |
| 183 | antithrombin | 4 | 0 | 0 | 4 | 4 | 0 |
| 184 | blood coagulation factor I | 4 | 0 | 1 | 3 | 4 | 0 |
| 185 | international normalized ratio (INR) | 3 | 0 | 0 | 3 | 3 | 0 |
| 186 | four blood coagulation indexes | 3 | 0 | 0 | 3 | 3 | 0 |
| 187 | partial thromboplastin time | 2 | 0 | 0 | 2 | 2 | 0 |
| 188 | thrombin time (TT) | 2 | 0 | 0 | 2 | 2 | 0 |
| 189 | coagulation function | 1 | 0 | 0 | 1 | 1 | 0 |
| 190 | prothrombin activity | 1 | 0 | 0 | 1 | 1 | 0 |
| 191 | five blood coagulation indexes | 1 | 0 | 0 | 1 | 1 | 0 |
| 192 | ferritin | 1 | 0 | 0 | 1 | 1 | 0 |
| 193 | transferrin saturation | 1 | 0 | 0 | 1 | 1 | 0 |
| 194 | folic acid | 1 | 0 | 0 | 1 | 1 | 0 |
| 195 | total iron binding capacity | 1 | 0 | 0 | 1 | 1 | 0 |
| 196 | malonaldehyde (MDA) | 31 | 1 | 1 | 29 | 30 | 1 |
| 197 | Content of serum lipid peroxidation (LPO) | 13 | 0 | 0 | 13 | 13 | 0 |
| 198 | Lactobacillus test | 4 | 0 | 0 | 4 | 4 | 0 |
| 199 | plasminogen activator inhibitor (PAI) | 3 | 0 | 0 | 3 | 3 | 0 |
| 200 | blood smear | 3 | 0 | 0 | 3 | 3 | 0 |
| 201 | Compliance of capacity(C1) | 2 | 0 | 0 | 2 | 1 | 1 |
| 202 | vitamin E | 2 | 0 | 0 | 2 | 2 | 0 |
| 203 | plasminogen activator inhibitor-I(PAI-1) | 2 | 0 | 0 | 2 | 2 | 0 |
| 204 | serum amylase (AMY) | 2 | 0 | 0 | 2 | 2 | 0 |
| 205 | Compliance of vibration(C2) | 2 | 0 | 0 | 2 | 1 | 1 |
| 206 | electrophoresis for detecting lipoprotein | 2 | 0 | 0 | 2 | 2 | 0 |
| 207 | adiponectin (APN) | 4 | 1 | 0 | 3 | 4 | 0 |
| 208 | enterococcus | 1 | 0 | 0 | 1 | 1 | 0 |
| 209 | the imbalance of prooxidant and antioxidant-(PAB) | 1 | 0 | 0 | 1 | 1 | 0 |
| 210 | elasticity index of artery | 1 | 0 | 0 | 1 | 1 | 0 |
| 211 | distensibility coefficient (DC) | 1 | 0 | 0 | 1 | 1 | 0 |
| 212 | Arterial compliance coefficient (CC) | 1 | 0 | 0 | 1 | 1 | 0 |
| 213 | Dihydrocholesterol | 1 | 0 | 0 | 1 | 1 | 0 |
| 214 | Stiffness index(β) | 1 | 0 | 0 | 1 | 1 | 0 |
| 215 | gluconic acid | 1 | 0 | 0 | 1 | 1 | 0 |
| 216 | leptin | 2 | 1 | 0 | 1 | 2 | 0 |
| 217 | bifidobacterium | 1 | 0 | 0 | 1 | 1 | 0 |
| 218 | Vitamin A | 1 | 0 | 0 | 1 | 1 | 0 |
| 219 | vitamin D | 1 | 0 | 0 | 1 | 1 | 0 |
| 220 | Angiotensin II | 1 | 0 | 0 | 1 | 1 | 0 |
| 221 | Elastic coefficient of pressure (Ep) | 1 | 0 | 0 | 1 | 1 | 0 |
| 222 | total antioxidant capacity | 1 | 0 | 0 | 1 | 1 | 0 |
| 223 | thromboxane B2(TXB2) | 14 | 0 | 0 | 14 | 14 | 0 |
| 224 | 8-isoprostanes | 1 | 0 | 0 | 1 | 1 | 0 |
| 225 | palpation on liver and spleen | 10 | 0 | 0 | 10 | 10 | 0 |
| 226 | fundus examination | 3 | 0 | 0 | 3 | 3 | 0 |
| 227 | iodothyronine | 1 | 0 | 0 | 1 | 1 | 0 |
| 228 | abdominal examination | 1 | 0 | 0 | 1 | 1 | 0 |
| 229 | cardiopulmonary auscultation | 1 | 0 | 0 | 1 | 1 | 0 |
| 230 | Length of thrombosis | 3 | 0 | 0 | 3 | 3 | 0 |
| 231 | Length of thrombosis in vitro | 2 | 0 | 0 | 2 | 2 | 0 |
| 232 | dry weight of thrombosis | 2 | 0 | 0 | 2 | 2 | 0 |
| 233 | wet weight of thrombosis | 2 | 0 | 0 | 2 | 2 | 0 |
| 234 | bulbar conjunctival color microcirculation test | 2 | 0 | 0 | 2 | 2 | 0 |
| 235 | Microcirculation retention time (MST) | 2 | 0 | 0 | 2 | 2 | 0 |
| 236 | state of ansae surrounding | 1 | 0 | 0 | 1 | 1 | 0 |
| 237 | form of venous plexus ansae | 1 | 0 | 0 | 1 | 1 | 0 |
| 238 | Nail fold microcirculation | 1 | 0 | 0 | 1 | 1 | 0 |
| 239 | Skin temperature | 1 | 0 | 0 | 1 | 1 | 0 |
| 240 | Microcirculatory blood flow | 1 | 0 | 0 | 1 | 1 | 0 |
| 241 | circulation retention time (NST) | 1 | 0 | 0 | 1 | 1 | 0 |
| 242 | Number of abnormal vessels | 1 | 0 | 0 | 1 | 1 | 0 |
| 243 | Number of normal vessels | 1 | 0 | 0 | 1 | 1 | 0 |
| 244 | transformation rate of tritium labeled thymidine lymphocyte | 2 | 0 | 0 | 2 | 2 | 0 |
| 245 | Erythrocyte-C3b receptor rosette rate | 3 | 0 | 0 | 3 | 3 | 0 |
| 246 | Erythrocyte immune promoting factor rosette rate | 1 | 0 | 0 | 1 | 1 | 0 |
| 247 | Erythrocyte immunosuppressive factor rosette rate | 1 | 0 | 0 | 1 | 1 | 0 |
| 248 | Rosette rate of erythrocyte circulating immune complex | 1 | 0 | 0 | 1 | 1 | 0 |
| 249 | ECG | 565 | 3 | 0 | 562 | 558 | 7 |
| 250 | Cardiac rhythm | 57 | 0 | 0 | 57 | 57 | 0 |
| 251 | Creatine Kinase (CK) | 232 | 3 | 0 | 229 | 231 | 1 |
| 252 | Heart function | 47 | 1 | 0 | 46 | 47 | 0 |
| 253 | Lactate Dehydrogenase (LDH) | 4 | 0 | 0 | 4 | 4 | 0 |
| 254 | Creatine Kinase Isoenzyme (CK-MB) | 4 | 0 | 0 | 4 | 4 | 0 |
| 255 | Heart enzyme | 1 | 0 | 0 | 1 | 1 | 0 |
| 256 | Estradiol(E2) | 4 | 0 | 0 | 4 | 4 | 0 |
| 257 | Testosterone(T) | 2 | 0 | 0 | 2 | 2 | 0 |
| 258 | Prolactin (PRL) | 1 | 0 | 0 | 1 | 1 | 0 |
| 259 | Luteal Hormone (LH) | 1 | 0 | 0 | 1 | 1 | 0 |
| 260 | luteinizing hormone | 1 | 0 | 0 | 1 | 1 | 0 |
| 261 | Progesterone(P) | 1 | 0 | 0 | 1 | 1 | 0 |
| 262 | Follicule-Stimulating Hormone (FSH) | 1 | 0 | 0 | 1 | 1 | 0 |
| 263 | Carban dioxide-combining Power | 8 | 0 | 0 | 8 | 8 | 0 |
| 264 | Partial pressure of oxygen | 2 | 0 | 0 | 2 | 2 | 0 |
| 265 | blood glucose | 405 | 1 | 0 | 404 | 404 | 1 |
| 266 | fasting blood-glucose | 74 | 2 | 0 | 72 | 73 | 1 |
| 267 | glycosylated hemoglobin (HbA1c) | 8 | 1 | 0 | 7 | 8 | 0 |
| 268 | postprandial blood glucose | 5 | 0 | 0 | 5 | 4 | 1 |
| 269 | glucose | 3 | 0 | 0 | 3 | 3 | 0 |
| 270 | glycometabolism | 2 | 0 | 0 | 2 | 2 | 0 |
| 271 | Oral Glucose Tolerance Test (OGTT) | 1 | 0 | 0 | 1 | 1 | 0 |
| 272 | platelet aggregation rate (AGG) | 36 | 0 | 1 | 35 | 34 | 2 |
| 273 | platelet adhesion rate (PAD) | 14 | 0 | 0 | 14 | 13 | 1 |
| 274 | p-selection | 7 | 0 | 1 | 6 | 7 | 0 |
| 275 | Platelet depolymerization rate | 2 | 0 | 0 | 2 | 2 | 0 |
| 276 | platelet membrane proteins -140(GMP-140) | 2 | 0 | 0 | 2 | 2 | 0 |
| 277 | Content of platelet α granular membrane protein | 1 | 0 | 0 | 1 | 1 | 0 |
| 278 | Membrane markers of platelet activation CD62p | 2 | 1 | 0 | 1 | 2 | 0 |
| 279 | Membrane markers of platelet activation CD63 | 3 | 1 | 0 | 2 | 3 | 0 |
| 280 | Platelet derived growth factor (BB) | 1 | 0 | 0 | 1 | 1 | 0 |
| 281 | blood pressure | 362 | 1 | 0 | 361 | 357 | 5 |
| 282 | Ankle brachial index(ABI) | 1 | 0 | 0 | 1 | 1 | 0 |
| 283 | Plasma viscosity(ηp) | 265 | 1 | 2 | 262 | 263 | 2 |
| 284 | Whole blood high shear viscosity | 194 | 2 | 2 | 190 | 194 | 0 |
| 285 | Whole blood low shear viscosity | 188 | 2 | 2 | 184 | 187 | 1 |
| 286 | Fibrinogen (Fb) | 149 | 0 | 1 | 148 | 147 | 2 |
| 287 | Whole blood viscosity(ηb) | 106 | 1 | 0 | 105 | 104 | 2 |
| 288 | Erythrocyte Aggregation Index (VAI) | 53 | 0 | 0 | 53 | 53 | 0 |
| 289 | Reduced viscosity(ηr) | 45 | 0 | 0 | 45 | 45 | 0 |
| 290 | hemorheology | 34 | 1 | 0 | 33 | 33 | 1 |
| 291 | Erythrocyte deformation coefficient (TK) | 18 | 0 | 0 | 18 | 18 | 0 |
| 292 | Erythrocyte Electrophoresis Rate | 16 | 0 | 0 | 16 | 16 | 0 |
| 293 | Whole blood medium shear viscosity | 14 | 0 | 1 | 13 | 14 | 0 |
| 294 | Erythrocyet index of rigidity (IR) | 12 | 0 | 0 | 12 | 12 | 0 |
| 295 | Erythrocyte Electrophoresis Time | 9 | 0 | 0 | 9 | 8 | 1 |
| 296 | Hematocrit | 8 | 0 | 0 | 8 | 8 | 0 |
| 297 | Thrombus Forming coefficient (TFL) | 5 | 0 | 0 | 5 | 5 | 0 |
| 298 | Erythrocyte Sclerosis Index | 4 | 0 | 0 | 4 | 4 | 0 |
| 299 | Max erythrocyte aggregation index | 4 | 0 | 0 | 4 | 4 | 0 |
| 300 | Hemocyte aggregation index (VAI) | 4 | 0 | 0 | 4 | 4 | 0 |
| 301 | relative viscosity | 3 | 0 | 0 | 3 | 3 | 0 |
| 302 | cholesterol crystal | 1 | 0 | 0 | 1 | 0 | 1 |
| 303 | Erythrocyte electrophoresis index | 1 | 0 | 0 | 1 | 1 | 0 |
| 304 | ndex of Filtration (IF) | 1 | 0 | 0 | 1 | 1 | 0 |
| 305 | Erythrocyte cylindrical lineation | 1 | 0 | 0 | 1 | 0 | 1 |
| 306 | max erythrocyte deformation index | 1 | 0 | 0 | 1 | 1 | 0 |
| 307 | TC | 3185 | 14 | 74 | 3097 | 3113 | 72 |
| 308 | TG | 3211 | 12 | 94 | 3105 | 3138 | 73 |
| 309 | HDL-C | 2709 | 17 | 63 | 2629 | 2646 | 63 |
| 310 | LDL-C | 2240 | 9 | 79 | 2152 | 2189 | 51 |
| 311 | Atherogenic Index (AI) | 223 | 1 | 5 | 217 | 215 | 8 |
| 312 | blood lipid | 42 | 0 | 0 | 42 | 39 | 3 |
| 313 | VLDL-C | 41 | 1 | 4 | 36 | 41 | 0 |
| 314 | oxidized low density lipoprotein (Ox-LDL) | 20 | 0 | 0 | 20 | 20 | 0 |
| 315 | TC/HDL-C | 15 | 0 | 0 | 15 | 14 | 1 |
| 316 | LDL-C/HDL-C (CHDindex) | 8 | 0 | 0 | 8 | 8 | 0 |
| 317 | HDL-C/LDL-C | 7 | 0 | 0 | 7 | 7 | 0 |
| 318 | HDL-C/TC | 6 | 0 | 0 | 6 | 6 | 0 |
| 319 | non-high-density lipoprotein cholesterol | 8 | 2 | 1 | 5 | 8 | 0 |
| 320 | full set ofblood lipid | 4 | 0 | 0 | 4 | 4 | 0 |
| 321 | HDL2-C | 2 | 0 | 0 | 2 | 2 | 0 |
| 322 | mean change percentage of LDL-C after treatment | 2 | 0 | 2 | 0 | 2 | 0 |
| 323 | HDL0-C | 1 | 0 | 0 | 1 | 1 | 0 |
| 324 | HDL2-C/HDL3-C | 1 | 0 | 0 | 1 | 1 | 0 |
| 325 | HDL4-C | 1 | 0 | 0 | 1 | 1 | 0 |
| 326 | lipoprotein except HDL-C | 1 | 0 | 0 | 1 | 1 | 0 |
| 327 | decline time of LDL-C | 1 | 0 | 0 | 1 | 1 | 0 |
| 328 | mean change percentage of TC from baseline to post treatment | 1 | 1 | 0 | 0 | 1 | 0 |
| 329 | decline time of TC | 1 | 0 | 0 | 1 | 1 | 0 |
| 330 | mean change percentage of TG from baseline to post treatment | 1 | 1 | 0 | 0 | 1 | 0 |
| 331 | decline time of TG | 1 | 0 | 0 | 1 | 1 | 0 |
| 332 | normalization time of lipid level | 1 | 0 | 0 | 1 | 1 | 0 |
| 333 | Blood lipid (4 items) | 1 | 0 | 0 | 1 | 1 | 0 |
| 334 | Blood lipid (5 items) | 1 | 0 | 0 | 1 | 1 | 0 |
| 335 | Lipid comprehensive index | 1 | 0 | 0 | 1 | 1 | 0 |
| 336 | Subtypes LDL | 1 | 0 | 0 | 1 | 1 | 0 |
| 337 | lipoprotein-α1 | 1 | 0 | 0 | 1 | 1 | 0 |
| 338 | lipoprotein-α2 | 1 | 0 | 0 | 1 | 1 | 0 |
| 339 | lipoprotein-α3 | 1 | 0 | 0 | 1 | 1 | 0 |
| 340 | lipoprotein-α4 | 1 | 0 | 0 | 1 | 1 | 0 |
| 341 | lipoprotein-α5 | 1 | 0 | 0 | 1 | 1 | 0 |
| 342 | lipid turbidity | 1 | 0 | 0 | 1 | 1 | 0 |
| 343 | Intermediate lipoProteinβ | 1 | 0 | 0 | 1 | 1 | 0 |
| 344 | C- Reactive Protein | 76 | 0 | 0 | 76 | 73 | 3 |
| 345 | interleukin- -6(IL-6) | 14 | 0 | 0 | 14 | 12 | 2 |
| 346 | tumor necrosis factor -α(TNF-α) | 15 | 0 | 0 | 15 | 13 | 2 |
| 347 | interleukin -8(IL-8) | 7 | 0 | 0 | 7 | 7 | 0 |
| 348 | matrix metalloproteinase (MMP-9) | 4 | 0 | 0 | 4 | 4 | 0 |
| 349 | Myocardial enzymes | 1 | 0 | 0 | 1 | 1 | 0 |
| 350 | Serum IGF-I | 1 | 1 | 0 | 0 | 1 | 0 |
| 351 | heart rate | 220 | 1 | 0 | 219 | 218 | 2 |
| 352 | weight | 214 | 6 | 1 | 207 | 210 | 4 |
| 353 | body mass index (BMI) | 83 | 9 | 3 | 71 | 76 | 7 |
| 354 | respiration | 51 | 1 | 0 | 50 | 51 | 0 |
| 355 | general items | 50 | 4 | 1 | 45 | 49 | 1 |
| 356 | pulse | 32 | 0 | 0 | 32 | 32 | 0 |
| 357 | temperature | 29 | 1 | 0 | 28 | 29 | 0 |
| 358 | height | 25 | 1 | 0 | 24 | 25 | 0 |
| 359 | physical examination | 18 | 1 | 0 | 17 | 18 | 0 |
| 360 | Waistline | 17 | 2 | 0 | 15 | 17 | 0 |
| 361 | waist hip ratio | 9 | 4 | 0 | 5 | 9 | 0 |
| 362 | hipline | 10 | 2 | 0 | 8 | 10 | 0 |
| 363 | Abdominal circumference | 7 | 0 | 0 | 7 | 7 | 0 |
| 364 | vital signs | 7 | 0 | 0 | 7 | 7 | 0 |
| 365 | Resting pulse rate | 1 | 0 | 0 | 1 | 1 | 0 |
| 366 | insulin | 5 | 0 | 0 | 5 | 5 | 0 |
| 367 | fasting insulin (FINS) | 4 | 0 | 0 | 4 | 4 | 0 |
| 368 | insulin sensitivity index | 3 | 0 | 0 | 3 | 3 | 0 |
| 369 | insulin resistance index(IR) | 2 | 0 | 0 | 2 | 2 | 0 |
| 370 | insulin resistance index | 1 | 0 | 0 | 1 | 1 | 0 |
| 371 | abdomen ultrasound | 39 | 0 | 0 | 39 | 36 | 3 |
| 372 | Chest X-ray | 12 | 0 | 0 | 12 | 12 | 0 |
| 373 | endothelium-dependent relaxing function of brachial artery (FMD) | 6 | 0 | 0 | 6 | 6 | 0 |
| 374 | cardiac uhrasonography | 4 | 0 | 0 | 4 | 4 | 0 |
| 375 | ultrasonic cardiogram | 3 | 0 | 0 | 3 | 3 | 0 |
| 376 | expansion degree of inner diameter of brachial artery | 2 | 0 | 0 | 2 | 2 | 0 |
| 377 | Change of cerebral blood flow | 2 | 0 | 0 | 2 | 2 | 0 |
| 378 | rheoencephalogram | 2 | 0 | 0 | 2 | 2 | 0 |
| 379 | inner diameter of brachial artery | 1 | 0 | 0 | 1 | 1 | 0 |
| 380 | Femoral ankle pulse wave velocity(faPWV) | 1 | 0 | 0 | 1 | 1 | 0 |
| 381 | Brachial artery pulse wave velocity (baPWV) | 1 | 0 | 0 | 1 | 1 | 0 |
| 382 | Carotid blood flow | 1 | 0 | 0 | 1 | 1 | 0 |
| 383 | Carotid-femoral artery pulse wave velocity(cfPWV) | 1 | 0 | 0 | 1 | 1 | 0 |
| 384 | Internal carotid artery blood flow | 1 | 0 | 0 | 1 | 1 | 0 |
| 385 | Carotid-radial artery pulse wave velocity(cbPWV) | 1 | 0 | 0 | 1 | 1 | 0 |
| 386 | Common carotid artery blood flow | 1 | 0 | 0 | 1 | 1 | 0 |
| 387 | skull CT | 1 | 0 | 0 | 1 | 1 | 0 |
| 388 | arteria dorsalis pedis ultrasound | 1 | 0 | 0 | 1 | 1 | 0 |
| 389 | Free Fatty Acid (FFA) | 3 | 0 | 0 | 3 | 3 | 0 |
| 390 | Phospholipid (PL) | 2 | 0 | 0 | 2 | 2 | 0 |
| 391 | Total lipids(TL) | 2 | 0 | 0 | 2 | 2 | 0 |
| 392 | thickness and area of the plaque in extracranial carotid artery | 1 | 0 | 0 | 1 | 1 | 0 |
| 393 | death | 3 | 2 | 1 | 0 | 3 | 0 |
| 394 | All-cause mortality | 1 | 1 | 0 | 0 | 1 | 0 |
| 395 | cardiovascular and cerebrovascular events | 10 | 1 | 3 | 6 | 10 | 0 |
| 396 | Erection function | 1 | 0 | 1 | 0 | 1 | 0 |
| 397 | quality of erection | 1 | 0 | 1 | 0 | 1 | 0 |
| 398 | angle of erection | 1 | 0 | 1 | 0 | 1 | 0 |
| 399 | clinical symptoms and signs | 295 | 9 | 0 | 286 | 246 | 49 |
| 400 | primary clinical symptoms and signs | 18 | 1 | 0 | 17 | 17 | 1 |
| 401 | Myopathic symptoms | 1 | 0 | 0 | 1 | 1 | 0 |
| 402 | muscle symptoms | 1 | 0 | 0 | 1 | 1 | 0 |
| 403 | remission time of clinical symptoms | 1 | 0 | 0 | 1 | 1 | 0 |
| 404 | organ function | 1 | 0 | 0 | 1 | 1 | 0 |
| 405 | somatic symptoms | 1 | 0 | 0 | 1 | 1 | 0 |
| 406 | aging index | 1 | 0 | 0 | 1 | 0 | 1 |
| 407 | pain degree | 1 | 1 | 0 | 0 | 1 | 0 |
| 408 | Efficacy of hearing recovery | 1 | 0 | 0 | 1 | 0 | 1 |
| 409 | remission rate of headache | 1 | 0 | 0 | 1 | 0 | 1 |
| 410 | gastrointestinal symptom | 1 | 0 | 0 | 1 | 1 | 0 |
| 411 | score of gastrointestinal symptoms | 1 | 1 | 0 | 0 | 1 | 0 |
| 412 | nitric oxide (NO) | 54 | 0 | 0 | 54 | 54 | 0 |
| 413 | endothelin (ET) | 39 | 1 | 2 | 36 | 39 | 0 |
| 414 | Soluble intercellular adhesion factor -1(sVCAM-1) | 15 | 0 | 2 | 13 | 13 | 2 |
| 415 | 6-keto-prostaglandin F1a | 13 | 0 | 0 | 13 | 13 | 0 |
| 416 | endothelin-1(ET-1) | 12 | 0 | 0 | 12 | 12 | 0 |
| 417 | Calcitonin gene-related peptide (CGRP) | 8 | 0 | 0 | 8 | 8 | 0 |
| 418 | Number of endothelial progenitor cells | 4 | 0 | 0 | 4 | 4 | 0 |
| 419 | vascular endothelial dilatation function | 4 | 0 | 0 | 4 | 4 | 0 |
| 420 | clonality | 1 | 0 | 0 | 1 | 1 | 0 |
| 421 | endothelial function | 1 | 0 | 0 | 1 | 1 | 0 |
| 422 | Endothelial leukocyte adhesion molecule(E-LAM) | 2 | 0 | 0 | 2 | 2 | 0 |
| 423 | Endothelial progenitor cell function | 1 | 0 | 0 | 1 | 1 | 0 |
| 424 | Adhesion ability of endothelial progenitor cells | 1 | 0 | 0 | 1 | 1 | 0 |
| 425 | Migration ability of endothelial progenitor cells | 1 | 0 | 0 | 1 | 1 | 0 |
| 426 | Proliferation ability of endothelial progenitor cells | 1 | 0 | 0 | 1 | 1 | 0 |
| 427 | prostacyclin | 1 | 0 | 0 | 1 | 1 | 0 |
| 428 | Stable metabolites of prostacyclin | 1 | 0 | 0 | 1 | 1 | 0 |
| 429 | Vascular endothelial growth factor(VEGF) | 3 | 0 | 0 | 3 | 3 | 0 |
| 430 | Nitric oxide synthase NOS) | 1 | 0 | 0 | 1 | 1 | 0 |
| 431 | Static balance of closed eyes | 1 | 0 | 0 | 1 | 1 | 0 |
| 432 | Detection of Infrared Radiation Heat of Meridian Points | 1 | 0 | 0 | 1 | 1 | 0 |
| 433 | Static balance of eye opening | 1 | 0 | 0 | 1 | 1 | 0 |
| 434 | vestibular function | 2 | 0 | 0 | 2 | 2 | 0 |
| 435 | Dysfunction items | 1 | 0 | 0 | 1 | 1 | 0 |
| 436 | cornea | 1 | 0 | 0 | 1 | 1 | 0 |
| 437 | crystalline lens | 1 | 0 | 0 | 1 | 1 | 0 |
| 438 | CM syndrome effect | 395 | 7 | 4 | 384 | 58 | 337 |
| 439 | CM syndrome score | 333 | 3 | 16 | 314 | 192 | 141 |
| 440 | tongue manifestation | 54 | 2 | 1 | 51 | 51 | 3 |
| 441 | pulse manifestation | 50 | 2 | 1 | 47 | 47 | 3 |
| 442 | Constitution score | 5 | 1 | 0 | 4 | 3 | 2 |
| 443 | CM syndrome effectiveness (synthesis) | 5 | 0 | 0 | 5 | 0 | 5 |
| 444 | CM syndrome efficacy (secondary symptom) | 4 | 1 | 0 | 3 | 3 | 1 |
| 445 | CM syndrome efficacy (primary symptom) | 4 | 1 | 0 | 3 | 3 | 1 |
| 446 | CM syndrome efficacy (single item) | 4 | 0 | 0 | 4 | 0 | 4 |
| 447 | CM symptom score | 3 | 1 | 0 | 2 | 2 | 1 |
| 448 | degree of CM syndrome | 1 | 1 | 0 | 0 | 1 | 0 |
| 449 | disappearing rate of CM syndrome | 1 | 0 | 0 | 1 | 0 | 1 |
| 450 | disappearing rate of primary symptom | 1 | 0 | 0 | 1 | 1 | 0 |
| 451 | cost | 2 | 2 | 0 | 0 | 2 | 0 |
| 452 | treatment regimen period | 1 | 0 | 0 | 1 | 1 | 0 |
